# Epidemiology of Antimicrobial Resistance Genes in *Staphyloccocus aureus* Isolates from a Public Database in a One Health Perspective—Sample Characteristics and Isolates’ Sources

**DOI:** 10.3390/antibiotics12071225

**Published:** 2023-07-24

**Authors:** Francesca Zaghen, Valerio Massimo Sora, Gabriele Meroni, Giulia Laterza, Piera Anna Martino, Alessio Soggiu, Luigi Bonizzi, Alfonso Zecconi

**Affiliations:** 1 Department of Biomedical, Surgical and Dental Sciences-One Health Unit, School of Medicine, University of Milan, Via Pascal 36, 20133 Milan, Italy; 2 Department of Clinical and Community Sciences, School of Medicine, University of Milan, Via Celoria 22, 20133 Milan, Italy

**Keywords:** *S. aureus*, One Health, antimicrobial resistance, molecular epidemiology

## Abstract

*Staphylococcus aureus* is considered one of the most widespread bacterial pathogens for both animals and humans, being the causative agent of various diseases like food poisoning, respiratory tract infections, nosocomial bacteremia, and surgical site and cardiovascular infections in humans, as well as clinical and subclinical mastitis, dermatitis, and suppurative infections in animals. Thanks to their genetic flexibility, several virulent and drug-resistant strains have evolved mainly due to horizontal gene transfer and insurgence of point mutations. Infections caused by the colonization of such strains are particularly problematic due to frequently occurring antibiotic resistance, particulary methicillin-resistant *S. aureus* (MRSA), and are characterized by increased mortality, morbidity, and hospitalization rates compared to those caused by methicillin-sensitive *S. aureus* (MSSA). *S. aureus* infections in humans and animals are a prime example of a disease that may be managed by a One Health strategy. In fact, *S. aureus* is a significant target for control efforts due to its zoonotic potential, the frequency of its illnesses in both humans and animals, and the threat posed by *S. aureus* antibiotic resistance globally. The results of an epidemiological analysis on a worldwide public database (NCBI Pathogen Detection Isolate Browser; NPDIB) of 35,026 *S. aureus* isolates were described. We considered the diffusion of antibiotic resistance genes (ARGs), in both human and animal setting, and the results may be considered alarming. The result of this study allowed us to identify the presence of clusters with specific ARG patterns, and that these clusters are associated with different sources of isolation (e.g., human, non-human).

## 1. Introduction

*Staphylococcus aureus* is considered one of the most widespread bacterial pathogens for both animals and humans, being a causative agent of various diseases like food poisoning, respiratory tract infections, nosocomial bacteremia, and surgical site and cardiovascular infections in humans, as well as clinical and subclinical mastitis, dermatitis, and suppurative infections in animals [1,2]. *S. aureus* has the ability to produce a variety of virulence factors that cause tissue injury, immune evasion, colonization, cell–cell interactions, and adhesion [3,4,5]. Due to their genetic flexibility, several virulent and drug-resistant strains have evolved mainly through horizontal gene transfer and insurgence of point mutations [6]. In particular, methicillin-resistant *S. aureus* (MRSA) is characterized by increased mortality, morbidity, and hospitalization rates compared to those caused by methicillin-sensitive *S. aureus* (MSSA) [7,8]. As stated in the latest ECDC (European Centre for Disease Prevention and Control) annual epidemiological report, MRSA strains have a population-weighted EU/EEA (Europena Union/European Economic Area) mean prevalence of 15.8% [9]. Over time, *S. aureus* evolved resistance to various antibiotics, including conventional betalactam antibiotics (e.g., penicillin and its derivatives) [10] as well as to the most recent ones such as vancomicyin [11]. In addition to specific antibiotic resistance, biofilm also contributes to nonspecific antibiotic resistance, which is a common feature in many biofilm-associated *S. aureus* infections [12].

*S. aureus* infections in humans and animals are a good example of a disease that may be managed by a One Health strategy. In fact, *S. aureus* is a significant target for control efforts due to its zoonotic potential, the frequency of illnesses caused in both humans and animals, and the threat posed by *S. aureus* antibiotic resistance globally. These programs should take into account the genetic and phenotypic traits of the bacteria, the epidemiology of the illness, and a strategy that takes into account isolates from both humans and animals as well as the possible risk related to bacteria and ARGs spreading via the environment. To the best of our knowledge, the literature lacks studies investigating the possible relation between molecular antibiotic resistance patterns and sources of bacterial isolation (species, clinical status, organ…). With the aim of contibuting to fill this gap, we present the results of an epidemiological analysis of a worldwide public database (NCBI Pathogen Detection Isolate Browser; NPDIB) including 35,026 *S. aureus* isolates to better characterize the origin of antibiotic-resistant isolates.

## 2. Results

### 2.1. Data Description

The public database at the date 30 April 2022 included 35,026 isolates; among them, 16,787 (47.9%) were classified as clinical and human-associated (HUA); 2091 (6.0%) were isolated from animals, farms, or environmental sources, and classified as non-human-associated (NHA); 15,355 (43.8%) had an unknown origin (UNK); and 793 (2.3%) were from other human sources. Due to the relative low frequency of isolates classified as other human sources and their heterogeneity, these latter isolates were not furthermore considered in the epidemiological analyses.

The total number of isolates after data polishing was brought to 34,233, of which 2091 (6.1%) isolates belonged to the NHA class, 16,787 (49.0%) belonged to the HUA class, and 15,355 (44.9%) belonged to the UNK class.

**Figure 1:**
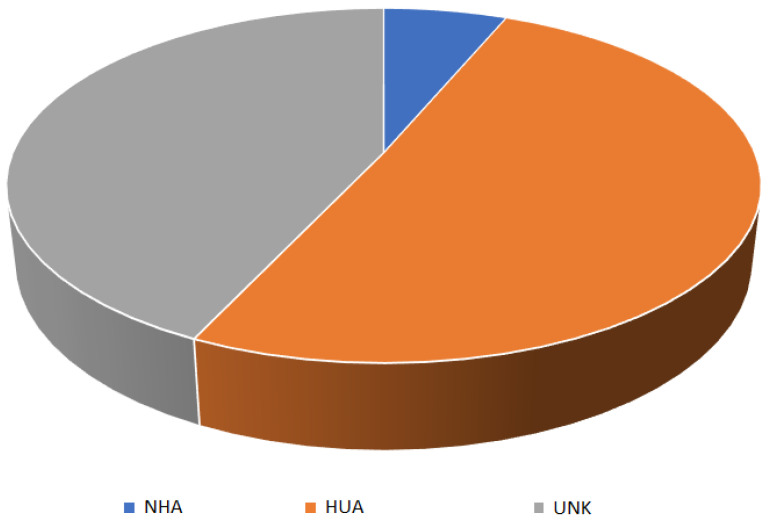
Distribution of *S. aureus* isolates by source (NHA = non-human-associated, HUA = human associated, UNK = unknown origin).

Table 1 and Figure 1 report the distribution of the sources of the isolates classified by the three categories considered (clinical, animal/farms/environment and unknown).

### 2.2. Resistance Gene Distribution

*S. aureus* is a well-known pathogen that carries a wide variety of ARGs. The database reported 67 different ARGs, and in this study, we considered 39 ARGs, excluding the ones having a prevalence <2%. The frequencies ranged from 2% of *dfrS1* to 99.9% of *mepA* (Table 2). We provided a detailed description of the antibiotic classes’ resistance and related mechanism of all the ARGs defined in the database in Supplementary Table S1.

The resistance genes shown in Table 2 belong to 13 different classes of antibiotics: tetracyclines, penams, phosphonic acid, fluoroquinolones, aminoglycosides, nucleosides, macrolides, glycopeptides, diaminopyrimidines, rifamycines, mupirocines, phenicols, and fusidanes. *BlaI*, *blaR1*, *mecR1*, and *mecI* genes were excluded from the study since they are regulatory genes of *blaZ* and *mecA,* respectively.

Regarding ARG diffusion, we identified the most widespread ARG for each antibiotic class: among the ARGs for amynoglycosides, the gene with the highest frequency is *ant(9)-la*, with a total of 9179 (17.8%) positive isolates; *dfrG* for diaminopyrimidines, with 3657 (10.7%) positive isolates; *parC* for the fluoroquinolones class, with 17,446 (51%) positive isolates; *mup(A)* for the mupirocines class, with 1421 (4.2%) positive isolates; *mecA* for the penams class (67.1%). The relative frequencies for *fex(A)* and *catA*, belonging to the phenicols class, are 806 (2.4%) and 795 (2.3%) positive isolates, respectively; in the phosphonic acid class, *fos(B)* is the most prevalent gene, with 21,257 (62.1%) positive isolates; *erm(C)* is the most frequent gene identified in the macrolides class, with 5685 (16.6%) cases of positivity; and finally, *mepA* is the most represented gene of the tetracyclines class, with 34,205 (99.9%) positive isolates.

### 2.3. Cluster Analysis

Cluster analysis was performed to identify possible AMR patterns, which can be considered the expression of the specific ARG asset of different isolates, thus allowing for the analysis of a potential relationship with other factors such as source, geographical origin, and others. The analysis identified seven different clusters based on the presence of the ARGs described in Table 2. Table 3 describes the composition of the clusters based on ARG frequencies and Table 4 contains information on isolation category and the total number of isolates per cluster.

In Table 3, we can see that the distribution of the frequencies of the most represented ARG in each cluster and for each antibiotic class, apart for *mepA*, is not homogeneous: among all clusters, cluster 1 has higher frequencies for *dfrG*, *parC*, *fex(A)*, and *erm(C)*; cluster 2 does not have high frequencies for any of the considered ARGs; cluster 3 shows an abundance of *fusC*; cluster 4 is characterized by high frequencies of the genes *ant(9)-la*, *abc-f*, *bleO*, *sat4*, *mecA*, *fos(B),* and *rpoB*; cluster 5 has higher frequencies of *abc-f*, *bleO*, *sat4*, *mecA,* and *fos(B)*; in cluster 6, the more abundant ARGs are *ant(9)-la*, *dfrG*, *parC*, *mup(A)*, *mecA,* and *fosB;* and finally, cluster 7 is characterized by an higher frequency of the gene *fex(A)*. When specific genes were considered, *ant(9)-la* is concentrated mainly in cluster 4 and 6; *abc-f* in cluster 4 and 5; *dfrG* and *parC* are more prevalent in cluster 1 and 6; *fusC* is mostly predominant in cluster 3; *bleO* and *sat4* have higher frequencies in cluster 4 and 5; *mup(A)* has the highest prevalence in cluster 6; *mecA* has the highest prevalence in cluster 4, 5, and 6; *fex(A)* is nearly absent in all clusters apart from cluster 1 and 7; *fos(B)* has high frequencies throughout all clusters, particularly in cluster 4, 5, and 6; *rpoB* is concentrated in cluster 4 and 6; and finally, *erm(C)* has an high prevalence in cluster 1.

**Figure 2:**
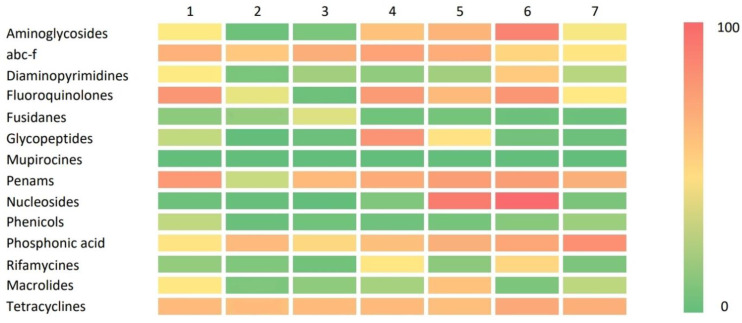
Graphical representation (heat map) of the antibiotic classes’ related ARG frequencies in each cluster.

In Figure 2, we tried to graphically represent the ARG rates divided for relative antibiotic class and clusters to visually describe the ARG distribution among all seven clusters.

**Figure 3:**
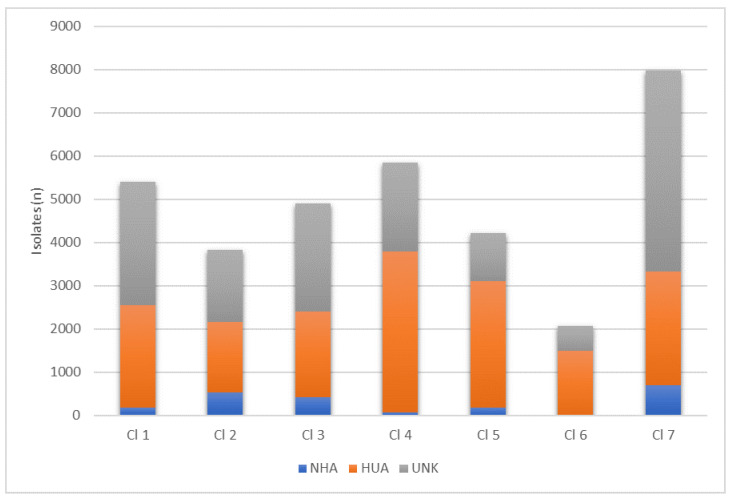
Distributions of isolates by source (NHA = non-human-associated, HUA = human-associated, UNK = unknown origin) among the seven clusters identified.

In Table 4 and Figure 3, we reported the distribution of the isolates among the clusters based on the isolation source. Clusters 4, 5, and 6 showed the highest frequency of HUA isolates with values >60%; clusters 2, 3, and 7 showed the highest frequencies of NHA isolates, even though this category is still not the predominant one in these clusters. The UNK category, including all the isolates that are not characterized regarding the source of isolation, comprises roughly 50% of all clusters apart from clusters 4, 5, and 6. The characteristics of the different clusters in relation to the isolation source classes of the isolates shown as clusters 2 and 7 have the highest proportion of NHA isolates (25.3% and 33.2% of total NHA isolates, respectively), while clusters 4 and 5 have the highest proportion of HUA isolates (22.2% and 17.4% of total HUA isolates, respectively). UNK isolates have the highest proportion in cluster 7 (30.2% of total UNK isolates). For all the clusters, a significant statistical difference was observed among the frequency of isolates classified by source.

## 3. Discussion

### 3.1. Relevance of the Dataset

The NPDIB database can be considered an important source of data, since it is an open access database that gathers information like date and source of isolation, geographical localization, and AMR genes’ presence of different bacterial isolates from all over the world. Thanks to its characteristics, it can be used as a reliable source of data to monitor AMR spread and perform epidemiological analyses with a statistical power that cannot be achieved through conventional methods. This information source does not precisely correspond to epidemiological guidelines (i.e., random sampling) because is based on voluntary upload of data. The remarkably high number of *S. aureus* isolates in the database, however, may be regarded as representative of the community of these bacteria linked to human or animal pathologies.

### 3.2. Antimicrobial Resistance Genes

Overall, 35 ARGs, divided into thirteen antibiotic classes, have been considered for the analyzes conducted in this study. The summary characteristics of these ARGs may be described as follows.

*ant(9)-la* is the gene responsible for the expression of aminoglycoside O-nucleotidyltransferase which catalyzes the adenylation of an AMP group from a substrate to the aminoglycoside molecule to make it inactive and convey specific resistance to spectinomycin [13,14].

*abc-f* is responsible for the expression of antibiotic resistance ABC-F proteins responsible for mediating resistance to a wide variety of antibiotics targeting the 50S ribosomal subunit by dissociating bound antibiotic molecules from the ribosome [15].

*dfrG* encodes the synthesis of dihydrofolate reductase and is mainly associated with trimethoprim resistance [16].

*S. aureus* resistance to fluoroquinolones comes from mutations in various genes, such as *parC*, resulting in the synthesis of altered proteins, in the quinolone resistance-determining region, which are less susceptible or insensible to this class of antibiotics [10,16].

*fusC* is responsible for the expression of FusC protein, which is a protein that actively protects the elongation factor-G from fusic acid molecules, enabling the pathogen to exert resistance to the fusidanes class of antibiotics [17].

Bleomycin and the related antibiotics phleomycin and tallysomycin function as DNA-breaking molecules capable of killing both procaryotic and eucaryotic cells at low concentrations. Plasmid-mediated resistance to bleomycin is widely spread among both clinically relevant Gram-negative and Gram-positive bacteria resistant to aminoglycosides [18]. The mechanism of resistance consists in the binding of the acidic bleomycin resistance proteins (BRPs), encoded by the *ble* genes, via electrostatic interactions to the bleomycin molecule, characterized by a basic pH, to prevent DNA cleavage [19].

The *mupA* gene, which is another name for the *ileS2* gene, is responsible for producing an isoleucyl-t-RNA synthetase that is resistant to mupirocin, an antibiotic that reversibly binds to the active site of bacterial and archaeal isoleucyl-t-RNA synthetase and competes with isoleucine and ATP or Ile-AMP [16,20].

*sat4* is a gene that confers resistance to streptothricin, an antibiotic that causes mRNA mistranslation and protein synthesis inhibition by interacting with the ribosome and encoding for the streptothricin acetyltransferase Sat4 [21].

The penicillin-binding proteins (PBPs) involved in peptidoglycan production are closely related to the mechanism of methicillin resistance. Additionally, MRSA strains produce PBP2A, which replaces PBP2’s transpeptidase activity and takes over the activity of other, inactivated PBPs. The PBP2 transpeptidase domain is made inactive by penams, but the PBP2 transglycosylase domain is still active, and in the case of MRSA strains, works in conjunction with the PBP2A transpeptidase to enable the synthesis of the cell wall [22].

The gene *fexA* encodes a protein of 475 amino acids with fourteen transmembrane domains, which represents an efflux protein of the major facilitator superfamily, FexA, able to actively remove chloramphenicol from the bacterial cell [23].

Fosfomycin is an inhibitor of peptidoglycan synthesis, and the cause of phosphomycin resistance in *S. aureus* is the synthesis of the metalloenzyme FosB, encoded by the *fosB* gene, which catalyzes the Mg_2_^+^-dependent attachment of L-cysteine to the phosphomycin ring [24].

Rifampicin inhibits transcription by interfering with the beta subunit of RNA polymerase. Resistance to rifampicin in *S. aureus* is determined by mutations in the *rpoB* gene encoding the B subunit of RNA polymerase. The most common are mutations that cause amino acid sequence changes in the RpoB protein, leading to a reduced affinity of the enzyme for the antibiotic [25].

The resistance mechanisms to macrolides are various in *S. aureus*. The most common one involves antibiotic’s target site modification, and it is carried out by the enzyme adenylyl-N-methyltransferase Erm (erythromycin ribosome methylation). The gene encoding Erm methylase synthetase may be expressed in a constitutive manner, in which case strains show resistance to all macrolides, or in an inducible manner, in which case resistance occurs only to antibiotics that are inducers of methylase synthesis. Resistance to the other macrolide-class antibiotics requires the presence of an inducer, which may be erythromycin or another macrolide. Inducible resistance to macrolides in *S. aureus* is most often determined by the *ermA* or *ermC* genes [26,27].

Tetracyclines inhibit protein synthesis by interfering with the 30S subunit of the ribosome, and the mechanism of resistance to tetracyclines in *S. aureus* usually involves active removal of the antibiotic from the bacterial cell and ribosomal protection. The MepA efflux pump, encoded by the *mepA* gene, is a part of the multidrug and toxic extrusion (MATE) family, and the decreased susceptibility to antibiotics (mainly fluoroquinolones, tetracycline), biocides, and dyes may indirectly be associated with overexpression of these pumps [16,28].

Despite concerns about the risk of an increasing frequency of vancomycin resistance, we observed frequencies below 2% (precisely 0.1% for *vanA*, 0.1% for *vanH-A*, 0.1% for *vanR-A*, 0.1% for *vanS-A*, 0.1% for *vanX-A*, 0.1% for *vanY-A*, and 0.1% for *vanZ-A*) for this antimicrobial molecule. The presence of vancomycin (VAN) resistance genes’ lowers the ability of the pathogen to spread because it restricts other biological functions of the bacterial cells, like being able to efficiently replicate and spread from one host to another; this biological concept can be referred to as “fitness cost” [29]. Indeed, several vancomycin-resistant (VRSA) and intermediate resistance to vancomycin (VISA) strains have appeared but have not spread throughout the population, making VAN still the antibiotic of last resort for MRSA infections [11,30]. These results partially support a recent work published by Wu and colleagues [31] stating that vancomycin resistance has increased globally in the past years, but overall frequency of resistant isolates can still be considered rather low.

### 3.3. Antimicrobial Resistance Genes’ Pattern

The different isolates may carry different distributions of AMR genes; therefore, the pattern of resistance may represent a more appropriate description of the features of the isolates in relation to human and animal diseases. The resistance patterns of the clusters shown as ARGs related to abc-f proteins, phosphonic acid, and tetracyclines are evenly spread across all clusters, which is in accordance with several studies investigating rates of resistance to these antibiotics [32,33,34]. Cluster 2 can be identified as the cluster with lowest presence of ARGs, while clusters 4, 5, and 6 have high rates of presence of ARGs related to the nine different antibiotic classes. Indeed, clusters 4, 5, and 6 have high rates of positivity for ARGs related to aminoglycosides, abc-f proteins, fluoroquinolones, penams, nucleosides, phosphonic acid, and tetracyclines, while high rates of ARGs related to glycopeptides and rifamycines characterize only cluster 4, high rates of ARGs related to glycopeptides and macrolides characterize only cluster 5, and high rates of ARGs related to diaminopyrimidines and rifamycines only characterize cluster 6. ARGs related to diaminopyrimidines, fusidanes, mupirocines, and phenicols have a low level of prevalence, with the only exception being diaminopyrimidines in cluster 6. The results related to mupirocines and phenicols are in accordance with other published studies [35,36], while the results that we obtained for diaminopyrimidines and fusidanes are discordant from what has been reported in the literature [37,38,39]. Penam-related ARGs are frequently observed in every cluster, with frequencies similar to the ones stated in the Global Antimicrobial Resistance and Use Surveillance System (GLASS) Report of 2022 [40], with the only exception of cluster 2, where the observed frequency is exceptionally low. Mupirocines and phenicols are the only two classes of antibiotics where the frequencies of the related ARGs can be considered nonrelevant in all clusters. Comparing the clusters’ ARG frequencies and the source of isolation composition, we observed that the clusters composed mainly by HUA isolates (cluster 4, 5 and 6) had higher frequencies of ARGs related to several different antibiotic classes, while clusters 2 and 7, characterized by a higher number of NHA isolates, show low-to-intermediate frequencies of ARG positivity. This information could be indicative of a different distribution of antibiotic resistance genetic elements between isolates from animals, food, the environment, and humans.

This database enables us to work with a superb amount of global data on ARG epidemiology. However, a potential weakness of this study is the fact that NHA isolates are far fewer than HUA and UNK isolates, so the imbalance among these categories could be a source of bias in the analysis. From a One Health perspective, the relative low frequency of isolates from animal sources should not be attributed to a low prevalence of illnesses in animals, but to a low frequency of upload of animal-related isolates, supporting the need to implement this database with more information on animal-derived isolates. Moreover, *S. aureus* has been known as an extremely important pathogen both in human and animal health [41,42], and the ability to study its AMR epidemiology with a One Health approach is of paramount importance to improve surveillance programs.

Finally, it should be also taken into account when interpreting these data that there are increasing evidences of genotype–phenotype discrepancies so that genomic AMR data should always be paired with phenotypic data, especially in clinical settings [43,44].

## 4. Materials and Methods

### 4.1. NCBI Pathogen Detection Isolate Browser and Antibacterial Data (NPDIB)

Approximately one million isolates from 53 different bacteria are currently available in the NCBI pathogen detection isolate browser (NPDIB). The parameters selected to perform an epidemiological study on *S. aureus* strains uploaded to this database were retrieved from a previous study [45]. Briefly, the data were exported into Microsoft Excel and the identification data were organized into columns in a matrix. Each AMR gene was associated with a column, which was filled with 1 if the gene was discovered in the sample and 0 if it was not. The information in the other columns were changed to align the formats and switch out text entries for numbers.

### 4.2. Statistical Analysis

All the data were analyzed on SPSS 28.0.1.1 (IBM Corp., Armonk, NY, USA, 2022). We applied χ2 test with Bonferroni adjustment to analyze the frequency distribution. Fisher’s exact test was applied instead of χ2 test when cell numerosity was below 6.

To classify isolates based on the different combination of AMR genes, cluster analysis was applied with the following parameters: squared Euclidean distance, Ward’s agglomeration method, and truncation at 20% of total distance [46]. Cluster analysis is a multivariate technique allowing one to group isolates based on the characteristics they possess (e.g., AMR genes).

## 5. Conclusions

The analysis that we performed on the NPDIB database of globally collected *S. aureus* isolates could represent a useful tool to constantly monitor the evolution of ARG spread throughout different countries and environments, perfectly following the One Health paradigm. The results of this study allowed us to identify the presence of clusters with specific ARGs pattern, and that these clusters may be associated to different sources of isolation (e.g., human, non-human). Indeed, cluster analysis allowed to identify the clusters with isolates with higher frequency of AMR genes, and to associate them with their source. The presence of a significant higher frequency of HUA isolates among the clusters with higher AMR pattern, suggests that these isolates have higher risks for human health, and the specific AMR pattern should be considered in presence of clinical outcome. Moreover, the large differences in the source of isolates among the different clusters suggest that the development of surveillance and/or preventive programs should consider these differences to increase the efficacy of these programs.

This latter result supports the importance of characterizing the isolates not only for the presence of gene of importance, but also for their source of isolation (species, organ…). Using a voluntary-based database obviously has its drawbacks, such as it does not follow strict epidemiological guidelines for collecting isolates and the incompleteness of the majority of the data, but even taking into account these critical points, it cannot be overlooked that useful information can be gathered by such a large amount of data. We strongly believe that the attention of both public health and veterinary authorities should focus on implementing the use of this database to further increase the quantity and quality of the uploaded data, making it a useful tool to better adjust surveillance plans and contrast the ever-growing threat of AMR worldwide.

## Figures and Tables

**Table 1:** Sources of isolates’ distribution.

Category	Source	Isolates (N)	Relative Frequency among Category (%)
Non-Human-Associated (NHA)	Animal	725	34.7
	Environment	256	12.2
	Farm	201	9.6
	Food	909	43.5
Human-Associated (HUA)	Blood	4298	25.6
	Respiratory sources	7366	43.9
	Skin	1338	8.0
	Wound	704	4.2
	Abscess	312	1.8
	Other districts	1846	11.0
	Other sources	923	5.5
Unknown (UNK)	Unknown sources	14,247	92.8
	Other districts	432	2.8
	Other sources	676	4.4

**Table 2:** Antibiotic resistance genes’ frequencies (%) of the NPDIB isolates (threshold for prevalence >2%) ^1^.

Gene	TOTAL	NHA ^2^	HUA	UNK
*mepA*	99.9	100.0 ^a3^	100.0 ^b^	99.9 ^a^
*tet (38)*	99.0	99.4 ^a^	98.4 ^b^	99.6 ^a^
*blaI*	82.1	72.8 ^a^	80.5 ^b^	85.0 ^c^
*blaR1*	72.4	66.4 ^a^	75.4 ^b^	70.0 ^c^
*mecA*	67.1	49.9 ^a^	73.5 ^b^	62.4 ^c^
*blaZ*	66.7	66.4 ^a^	64.5 ^a^	69.2 ^b^
*fos(B)*	62.1	45.2 ^a^	71.4 ^b^	54.3 ^c^
*mecR1*	56.2	29.9 ^a^	66.7 ^b^	48.2 ^c^
*parC*	51.0	24.2 ^a^	59.0 ^b^	45.8 ^c^
*gyrA*	48.5	19.2 ^a^	57.1 ^b^	43.1 ^c^
*murA*	44.0	54.8 ^a^	43.0 ^b^	43.5 ^b^
*Abc-f*	40.4	34.3 ^a^	46.6 ^b^	34.4 ^a^
*glpT*	27.3	42.2 ^a^	20.4 ^b^	32.9 ^c^
*ant(9)-la*	26.8	6.4 ^a^	36.0 ^b^	19.5 ^c^
*erm(A)*	26.6	4.7 ^a^	35.9 ^b^	19.4 ^c^
*ant(6)-la*	21.4	24.8 ^a^	28.8 ^b^	12.7 ^c^
*mecI*	20.7	4.0 ^a^	30.2 ^b^	12.5 ^c^
*aadD1*	20.5	12.1 ^a^	28.6 ^b^	12.9 ^a^
*aph(3′)-lla*	20.4	18.1 ^a^	27.9 ^b^	12.5 ^c^
*aac(6′)-le/aph(2”)-la*	17.8	12.7 ^a^	18.1 ^b^	18.2 ^b^
*sat4*	17.5	8.9 ^a^	25.0 ^b^	10.5 ^a^
*erm(C)*	16.6	16.8 ^a^	15.4 ^b^	17.9 ^a^
*blaPC1*	16.5	9.9 ^a^	16.3 ^b^	17.6 ^c^
*bleO*	15.2	3.2 ^a^	21.5 ^b^	9.9 ^c^
*Tet(K)*	14.2	19.5 ^a^	10.9 ^b^	17.1 ^c^
*msr(A)*	13.4	9.1 ^a^	19.7 ^b^	7.0 ^c^
*mph(C)*	12.9	9.0 ^a^	19.2 ^b^	6.5 ^c^
*dfrG*	10.7	14.3 ^a^	11.4 ^b^	9.4 ^c^
*tet(M)*	10.6	11.4 ^a^	8.2 ^b^	13.1 ^a^
*parE*	6.2	1.2 ^a^	6.3 ^b^	6.8 ^b^
*rpoB*	5.3	3.9 ^a^	6.6 ^b^	4.1 ^a^
*mup(A)*	4.2	0.5 ^a^	6.0 ^b^	2.6 ^c^
*tet(L)*	3.2	8.8 ^a^	2.4 ^b^	3.2 ^c^
*erm(B)*	3.0	12.7 ^a^	2.2 ^b^	2.5 ^b^
*ileS*	2.9	0.4 ^a^	2.8 ^b^	3.4 ^c^
*fex(A)*	2.4	9.8 ^a^	2.2 ^b^	1.5 ^c^
*fusC*	2.4	0.2 ^a^	2.1 ^b^	2.9 ^c^
*catA*	2.3	4.4 ^a^	2.9 ^b^	1.5 ^c^
*dfrS1*	2.0	1.0 ^a^	2.3 ^b^	1.8 ^c^

^1^ <2% aac(6′)-le; aph(2″)-l; apmA; cat-TC; cfr; dfrB; dfrF; dfr(K); erm(T); fosD; fosY; fus(A); fusB; lnu(A); mecC; mprF; rpIC_G152D; spd; tet(C); vanA; vanH-A; vanR-A; vanS-A; vanX-A; vanY-A; vanZ-A; vga(A); walK. ^2^ NHA = non-human-associated, HUA = human-associated, UNK = unknown origin. ^3^ values with different superscript among lines statistically differ at χ^2^ test or Fisher’s exact test (α = 0.05).

**Table 3:** ARG frequencies according to the antibiotic class and cluster; *n* = number of positive isolates. The genes with the highest frequency for each antibiotic class are represented in bold types.

Antibiotic Class	Gene	Cluster						
	ARG (*n*)	1	2	3	4	5	6	7
aminoglycosides	***ant(9)-la* (9179)**	**0.3%**	**0.3%**	**0.1%**	**95.7%**	**20%**	**94.7%**	**9.4%**
	*ant(6)-la* (7310)	10.6%	0.1%	0%	0.3%	94.3%	99.2%	8.7%
	*aadD1* (7033)	17.7%	1.1%	3.3%	70.5%	19.9%	23.6%	5.3%
	*aph(3′)-lla* (6984)	0.7%	0.5%	0.2%	2.9%	96.6%	99.3%	7.9%
	*aac(6′)-le/aph(2”)-la* (6102)	18.3%	1.7%	4%	23.3%	10.6%	92.1%	14.3%
antibiotics targeting protein synthesis	***Abc-f* (13,816)**	**49.7%**	**34.2%**	**52.1%**	**59.3%**	**53.2%**	**24.3%**	**13.3%**
diaminopyrimidines	***dfrG* (3657)**	**16.4%**	**2.2%**	**6.2%**	**0.8%**	**6.9%**	**64.2%**	**9%**
	*dfrS1* (681)	2.7%	0.6%	1.4%	4.9%	0.7%	1.1%	1.3%
fluoroquinolones	***parC* (17,446)**	**97.7%**	**13.3%**	**1.4%**	**92.2%**	**64.6%**	**96.0%**	**18.7%**
	*gyrA* (16,601)	95.0%	10.3%	0.2%	91.4%	61.1%	95.8%	14.6%
	*parE* (2126)	15.2%	0.8%	0.3%	13.5%	3.7%	14.3%	0.3%
fusidanes	***fusC* (807)**	**2.7%**	**3.1%**	**7.6%**	**0.9%**	**1.2%**	**0.7%**	**0.7%**
glycopeptides	***bleO* (5197)**	**5.9%**	**0.1%**	**0.6%**	**70.1%**	**16.2%**	**1%**	**0.6%**
mupirocines	***mup(A)* (1421)**	**5%**	**1.1%**	**1.5%**	**4.0%**	**7.0%**	**23.1%**	**0.4%**
	*ileS* (1000)	1.6%	0.4%	0.1%	11.9%	1.5%	4.9%	0.4%
nucleosides	***sat4* (5986)**	**0.7%**	**0.3%**	**0%**	**1.8%**	**87.2%**	**98.7%**	**1.4%**
penams	***mecA* (22,968)**	**94.4%**	**18.2%**	**32.0%**	**93.3%**	**91.3%**	**98.2%**	**53.5%**
	*blaZ* (22,850)	96.1%	0.4%	99.4%	56.5%	97.6%	27.1%	60.1%
	*blaPC1* (5641)	7.9%	0.1%	0.2%	10.7%	1.7%	66.2%	39.3%
phenicols	***fex(A)* (806)**	**10%**	**0.4%**	**0.8%**	**0%**	**0%**	**0%**	**2.6%**
	*catA* (795)	1.5%	0.4%	0.9%	1.6%	2.3%	4.6%	4.6%
phosphonic acid	***fos(B)* (21,257)**	**33.1%**	**51%**	**64.2%**	**96.9%**	**93.3%**	**99.5%**	**34.1%**
	*murA* (15,048)	10.3%	48.4%	5.5%	16.1%	57.6%	73.3%	93.8%
	*glpT* (9354)	0.3%	34.3%	1.7%	8%	2%	0.3%	92.6%
rifamycines	***rpoB* (1819)**	**3.1%**	**1.8%**	**0.9%**	**13.5%**	**2.5%**	**25.1%**	**1.6%**
macrolides	*mph(C)* (4401)	4.2%	0.6%	0.6%	6.2%	87.7%	1.9%	0.3%
	*msr(A)* (4571)	4.4%	0.9%	1.5%	6.4%	87.8%	2.1%	1.3%
	*erm(A)* (112)	0.3%	0.2%	0.1%	95.6%	19.9%	95.2%	8.5%
	***erm(C)* (5685)**	**50.7%**	**4.8%**	**10.5%**	**7.8%**	**11.4%**	**3.3%**	**15.7%**
	*erm(B)* (1015)	2.5%	0.9%	1%	0%	0.2%	0%	9.8%
tetracyclines	*tet (38)* (33,893)	99.6%	99.9%	100%	99.6%	93.9%	98.5%	100%
	*tet(k)* (4869)	8.5%	3.4%	9.1%	3.5%	8.4%	40.2%	30.7%
	*tet (L)* (1087)	9.9%	0.3%	0.7%	0.3%	2.6%	0.1%	4.7%
	*tet(m)* (3621)	1.1%	1.6%	1.6%	11.9%	1%	43.9%	22.3%
	***mepA* (34,205)**	**99.9%**	**100%**	**99.9%**	**100%**	**100%**	**100%**	**99.8%**

**Table 4:** Distribution of the isolates among clusters based on source (human, animal, and unknown).

Source of the Isolates	Cluster						
	1	2	3	4	5	6	7
NHA ^1^	189 (3.5%) ^a2^	528 (13.8%) ^a^	417 (8.5%) ^a^	66 (1.1%) ^a^	192 (4.6%) ^a^	4 (0.2%) ^a^	695 (8.7%) ^a^
HUA	2361 (43.8%) ^b^	1643 (43%) ^b^	1993 (40.6%) ^b^	3735 (63.8%) ^b^	2916 (69.1%) ^b^	1497 (72.5%) ^b^	2642 (33.1%) ^b^
UNK	2845 (52.7%) ^c^	1653 (43.2%) ^c^	2494 (50.9%) ^c^	2050 (35%) ^c^	1110 (26.3%) ^c^	564 (27.3%) ^c^	4639 (58.2%) ^c^
TOT	5395 (100%)	3824 (100%)	4904 (100%)	5851 (100%)	4218 (100%)	2065 (100%)	7976 (100%)

^1^ NHA = non-human-associated, HUA = human-associated, UNK = unknown origin, TOT = total. ^2^ values with different superscript among columns statistically differ (α = 0.05).

## Data Availability

Publicly available datasets were analyzed in this study. This data can be found here: https://www.ncbi.nlm.nih.gov/pathogens/isolates/#taxgroup_name:%22Staphylococcus%20aureus%22 (accessed on 30 April 2022).

## Supplementary Materials

The following supporting information can be downloaded at: https://www.mdpi.com/article/10.3390/antibiotics12071225/s1. Table S1: drug class resistance, resistance mechanism induced by antibiotic resistance genes and AMR gene family of the genes found in *S. aureus* isolates of NPDIB.
