# Supplementary material for: Epidemiology of Antimicrobial Resistance Genes in Staphyloccocus aureus Isolates from a Public Database in a One Health Perspective—Sample Characteristics and Isolates’ Sources
Source: Antibiotics (Basel). 2023 Jul 24;12(7):1225. doi: 10.3390/antibiotics12071225 (PMC10376612; doi:10.3390/antibiotics12071225)
Supplement: Supplementary file 1 [file antibiotics-12-01225-s001.zip › antibiotics-2508142-supplementary.pdf]

Table S1: drug class resistance, resistance mechanism induced by antibiotic resistance genes and AMR gene family of the genes found in *S. aureus* isolates of NPDIB.

Gene	Drug class	Resistance Mechanism	AMR gene family
ant(9)-la	aminoglycoside antibiotic	antibiotic inactivation	ANT(9)
ant(6)-la	aminoglycoside antibiotic	antibiotic inactivation	ANT(6)
aadD1	aminoglycoside antibiotic	antibiotic inactivation	ANT(4')
aph(3′)-lla	aminoglycoside antibiotic	antibiotic inactivation	APH(3')
aac(6′)-le/aph(2″)-la	aminoglycoside antibiotic	antibiotic inactivation	aminoglycoside bifunctional resistance protein
aac(6′)-le	aminoglycoside antibiotic	antibiotic inactivation	AAC(6')
aph(2″)-l	aminoglycoside antibiotic	antibiotic inactivation	APH(2'')
apmA	aminoglycoside antibiotic	antibiotic inactivation	apm acetyltransferase
spd	aminoglycoside antibiotic	antibiotic inactivation	ANT(9)
Abc-f	antibiotics targeting protein synthesis	ribosomal antibiotic target protection	ABC-F subfamily ATP-binding cassette ribosomal protection
dfrG	diaminopyrimidine antibiotic	antibiotic target replacement	trimethoprim resistant dihydrofolate reductase dfr
dfrS1	diaminopyrimidine antibiotic	antibiotic target replacement	trimethoprim resistant dihydrofolate reductase dfr
dfrB	diaminopyrimidine antibiotic	antibiotic target replacement	trimethoprim resistant dihydrofolate reductase dfr
dfrF	diaminopyrimidine antibiotic	antibiotic target replacement	trimethoprim resistant dihydrofolate reductase dfr
dfr(K)	diaminopyrimidine antibiotic	antibiotic target replacement	trimethoprim resistant dihydrofolate reductase dfr
parC	fluoroquinolone antibiotic	antibiotic target alteration	fluoroquinolone resistant parC
gyrA	fluoroquinolone antibiotic	antibiotic target alteration	fluoroquinolone resistant gyrA
parE	fluoroquinolone antibiotic	antibiotic target alteration	fluoroquinolone resistant parE
fusC	fusidane antibiotic	antibiotic target protection	Target protecting FusB-type protein conferring resistance to Fusidic acid
fus(A)	fusidane antibiotic	antibiotic target alteration	antibiotic resistant fusA

fusB	fusidane antibiotic	antibiotic target alteration	Target protecting FusB-type protein conferring resistance to Fusidic acid
bleO	glycopeptide antibiotic	antibiotic inactivation	Bleomycin resistant protein
vanA	glycopeptide antibiotic	antibiotic target alteration	glycopeptide resistance gene cluster, Van ligase
vanH-A	glycopeptide antibiotic	antibiotic target alteration	vanH
vanR-A	glycopeptide antibiotic	antibiotic target alteration	vanR
vanS-A	glycopeptide antibiotic	antibiotic target alteration	vanS
vanX-A	glycopeptide antibiotic	antibiotic target alteration	vanX
vanY-A	glycopeptide antibiotic	antibiotic target alteration	vanY
vanZ-A	glycopeptide antibiotic	antibiotic target alteration	vanZ
lnu(A)	lincosamide antibiotic	antibiotic inactivation	lincosamide nucleotidyltransferase (LNU)
mph(C)	macrolide antibiotic	antibiotic inactivation	macrolide phosphotransferase (MPH)
mup(A)	mupirocin-like antibiotic	antibiotic target alteration	antibiotic-resistant isoleucyl-tRNA synthetase (ileS)
ileS	mupirocin-like antibiotic	antibiotic target alteration	antibiotic-resistant isoleucyl-tRNA synthetase (ileS)
sat4	nucleoside antibiotic	antibiotic inactivation	streptothricin acetyltransferase (SAT)
rpIC_G152D	oxalidinone antibiotic	ribosomal antibiotic target protection	*rplC* point mutation
cfr	oxazolidinone antibiotic, lincosamide antibiotic, streptogramin antibiotic, phenicol antibiotic	antibiotic target alteration	Cfr 23S ribosomal RNA methyltransferase
mec C	penam	antibiotic target replacement	methicillin resistant PBP2
blaI	penam (penicillins)	antibiotic inactivation	BlaZ beta-lactamase
blaR1	penam (penicillins)	antibiotic inactivation	BlaZ beta-lactamase
mecA	penam (penicillins)	antibiotic target replacement	methicillin resistant PBP2
blaZ	penam (penicillins)	antibiotic inactivation	BlaZ beta-lactamase
mecR1	penam (penicillins)	antibiotic target replacement	methicillin resistant PBP2
mecI	penam (penicillins)	antibiotic target replacement	methicillin resistant PBP2
blaPC1	penam (penicillins)	antibiotic inactivation	BlaZ beta-lactamase
mprF	peptide antibiotic	antibiotic target alteration	defensin resistant mprF
walK	peptide antibiotic	antibiotic target alteration	daptomycin resistant walK

fex(A)	phenicol antibiotic	antibiotic efflux	major facilitator superfamily (MFS) antibiotic efflux pump
catA	phenicol antibiotic	antibiotic inactivation	chloramphenicol acetyltransferase (CAT)
cat-TC	phenicol antibiotic	antibiotic inactivation	chloramphenicol acetyltransferase (CAT)
fos(B)	phosphonic acid antibiotic	antibiotic inactivation	fosfomycin thiol transferase
murA	phosphonic acid antibiotic	antibiotic inactivation	antibiotic-resistant murA transferase
glpT	phosphonic acid antibiotic	antibiotic inactivation	antibiotic-resistant glpT
fosD	phosphonic acid antibiotic	antibiotic inactivation	fosfomycin thiol transferase
fosY	phosphonic acid antibiotic	antibiotic inactivation	fosfomycin thiol transferase
rpoB	rifamycin antibiotic	antibiotic target alteration, antibiotic target replacement	rifamycin-resistant beta-subunit of RNA polymerase (rpoB)
msr(A)	streptogramin antibiotic, macrolide antibiotic, streptogramin B antibiotic	antibiotic target protection	msr-type ABC-F protein
erm(A)	streptogramin antibiotic, macrolide antibiotic, streptogramin B antibiotic, streptogramin A antibiotic, lincosamide antibiotic	antibiotic target alteration	Erm 23S ribosomal RNA methyltransferase
erm(C)	streptogramin antibiotic, macrolide antibiotic, streptogramin B antibiotic, streptogramin A antibiotic, lincosamide antibiotic	antibiotic target alteration	Erm 23S ribosomal RNA methyltransferase
erm(B)	streptogramin antibiotic, macrolide antibiotic, streptogramin B antibiotic, streptogramin A	antibiotic target alteration	Erm 23S ribosomal RNA methyltransferase

	antibiotic, lincosamide antibiotic		
vga(A)	streptogramin antibiotic, streptogramin A antibiotic, lincosamide antibiotic, pleuromutilin antibiotic	antibiotic target protection	vga-type ABC-F protein
erm(T)	streptogramin antibiotic, streptogramin B antibiotic, macrolide antibiotic, lincosamide antibiotic, streptogramin A antibiotic	antibiotic target alteration	Erm 23S ribosomal RNA methyltransferase
tet (38)	tetracycline antibiotic	antibiotic efflux	major facilitator superfamily (MFS) antibiotic efflux pump
Tet(K)	tetracycline antibiotic	antibiotic efflux	major facilitator superfamily (MFS) antibiotic efflux pump
tet(M)	tetracycline antibiotic	antibiotic target protection	tetracycline-resistant ribosomal protection protein
tet(L)	tetracycline antibiotic	antibiotic efflux	major facilitator superfamily (MFS) antibiotic efflux pump
tet(C)	tetracycline antibiotic	antibiotic efflux	major facilitator superfamily (MFS) antibiotic efflux pump
mepA	tetracycline antibiotic, glycylcycline	antibiotic efflux	multidrug and toxic compound extrusion (MATE) transporter
